# Supplementary material for: PowerBacGWAS: a computational pipeline to perform power calculations for bacterial genome-wide association studies
Source: Commun Biol. 2022 Mar 25;5:266. doi: 10.1038/s42003-022-03194-2 (PMC8956664; doi:10.1038/s42003-022-03194-2)
Supplement: Supplementary file 2 — Supplementary Information [file 42003_2022_3194_MOESM2_ESM.pdf]

Supplementary Materials for:

PowerBacGWAS: a computational pipeline to perform power calculations for bacterial genome-wide association studies.

Authors: Francesc Coll*, Theodore Gouliouris, Sebastian Bruchmann, Jody Phelan, Kathy E. Raven, Taane G Clark, Julian Parkhill, Sharon J. Peacock

*Corresponding author: francesc.coll@lshtm.ac.uk

This file includes:

Supplementary Figure 1. Pan-genome GWAS power calculations obtained using the phenotype-simulation approach

Supplementary Figure 2. Variant GWAS power calculations obtained using the phenotype-simulation approach

Supplementary Figure 3. Burden test GWAS power calculations obtained using the phenotype-simulation approach

Supplementary Figure 4. Steps and scripts used to implement *PowerBacGWAS*

Supplementary Table 1. Sample sizes required to detect AMR causal variants of different frequencies and effect sizes

Supplementary Table 2. Sample sizes required to detect causal genes of different frequencies and heritability values in a pan-genome GWAS

Supplementary Table 3. Sample sizes required to detect SNPs of different MAF and heritability values

Supplementary Table 4. Sample sizes required to detect causal SNPs of different MAF, effect sizes and number of homoplasies in a variant GWAS

Supplementary data files not included in this file:

Supplementary Data 1: isolate metadata including ENA accessions

Supplementary Data 2: source data of Figure 2

Supplementary Data 3: source data of Figure 3

Formatted: Line spacing: 1.5 lines

Formatted: Line spacing: 1.5 lines

Supplementary Figure 1 Pan-genome GWAS power calculations obtained using the phenotype-simulation approach

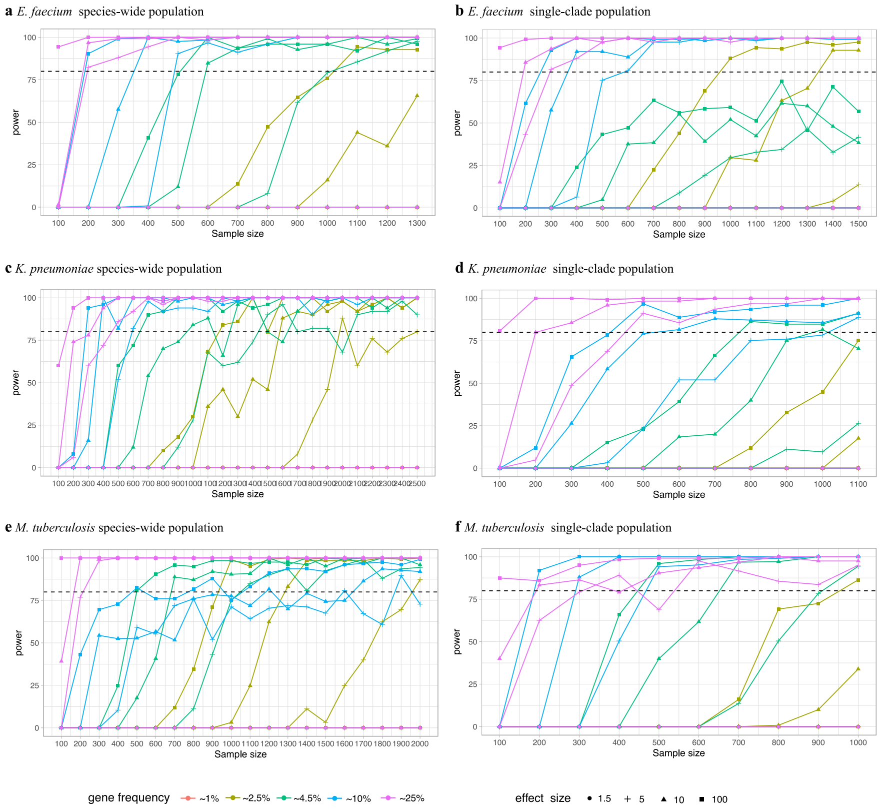

These plots show the sample sizes required to detect acquired genes of different effect sizes (in odds ratio units, showed as different point shapes) and gene frequencies (shown as different colours) in a pan-genome GWAS (binary phenotype, full heritability assumed), ~~in both species-wide and single-clade populations.~~ in both species-wide (panels a, c and e) and single-clade populations (panels b, d and f). The sample sizes required to detect genes of different effect sizes and frequencies with 80% power, extracted from these plots, are presented in Table 2. The y-axis shows the power, calculated as the proportion of GWAS replicates in which the causal gene is above the Bonferroni-corrected genome-wide significance threshold. The black and dotted horizontal line marks 80% power. Sample sizes are represented in the x-axis.

Supplementary Figure 2 Variant GWAS power calculations obtained using the phenotype-simulation approach

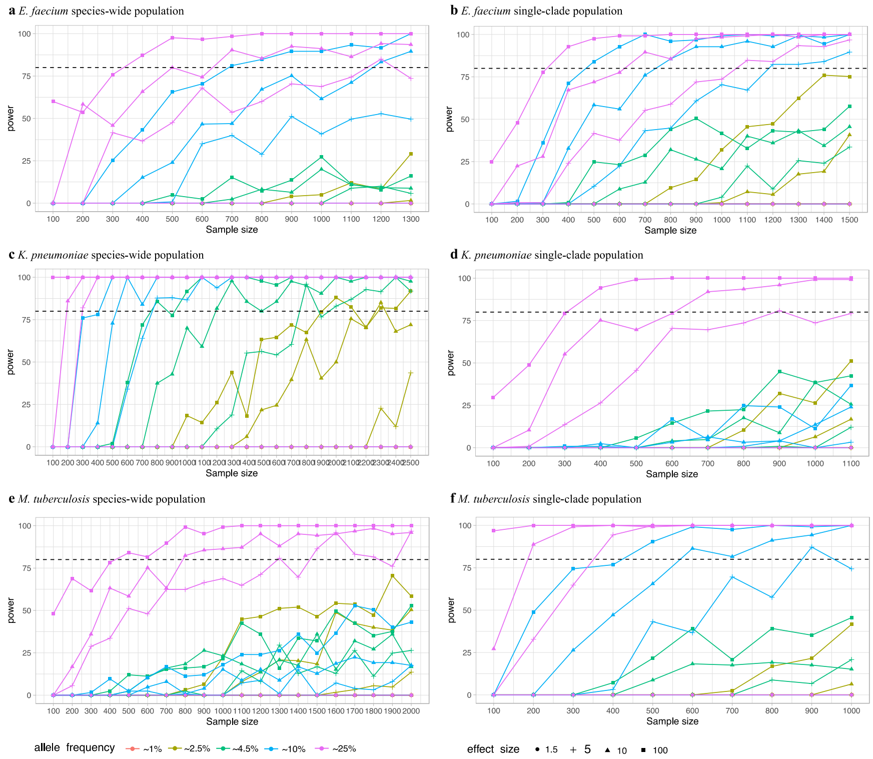

These plots show the sample sizes required to detect SNPs of different effect sizes (in odds ratio units, showed as different point shapes) and MAF (shown as different colours) in a variant GWAS (binary phenotype, full heritability assumed), in both species-wide (panels a, c and e) and single-clade populations (panels b, d and f) ~~in both species-wide and single-clade populations~~. The minimum sample sizes required to detect SNPs of different effect sizes and MAF with 80% power, extracted from these plots, are presented in Table 3. The y-axis shows the power, calculated as the proportion of GWAS replicates in which the causal SNP allele is above the Bonferroni-corrected genome-wide significance threshold. Sample sizes are represented in the x-axis.

Supplementary Figure 3 Burden test GWAS power calculations obtained using the phenotype-simulation approach

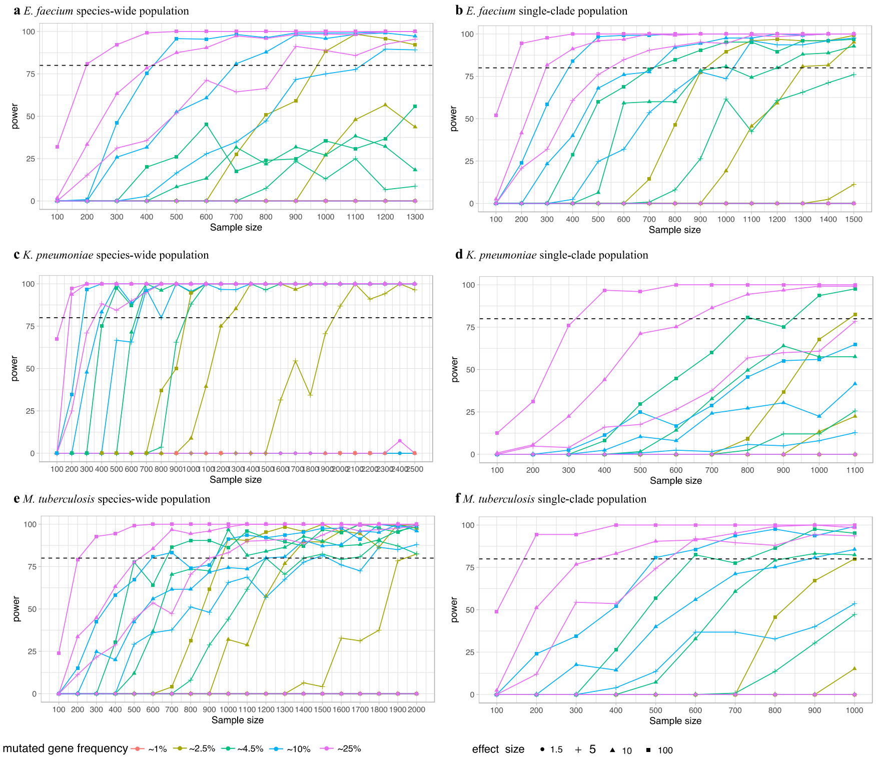

These plots show the sample sizes required to detect mutated genes of different effect sizes (in odds ratio units, showed as different point shapes) and mutated gene frequency (shown as different colours) in a burden test GWAS (binary phenotype, full heritability assumed),~~ in both species-wide and single-clade populations~~ in both species-wide (panels a, c and e) and single-clade populations (panels b, d and f). The minimum sample sizes required to detect genes of different effect sizes and frequencies with 80% power, extracted from these plots, are presented in Table 3. The y-axis shows the power, calculated as the proportion of GWAS replicates in which the causal mutated gene is above the Bonferroni-corrected genome-wide significance threshold. Sample sizes are represented in the x-axis.

Supplementary Figure 4 Steps and scripts used to implement *PowerBacGWAS*

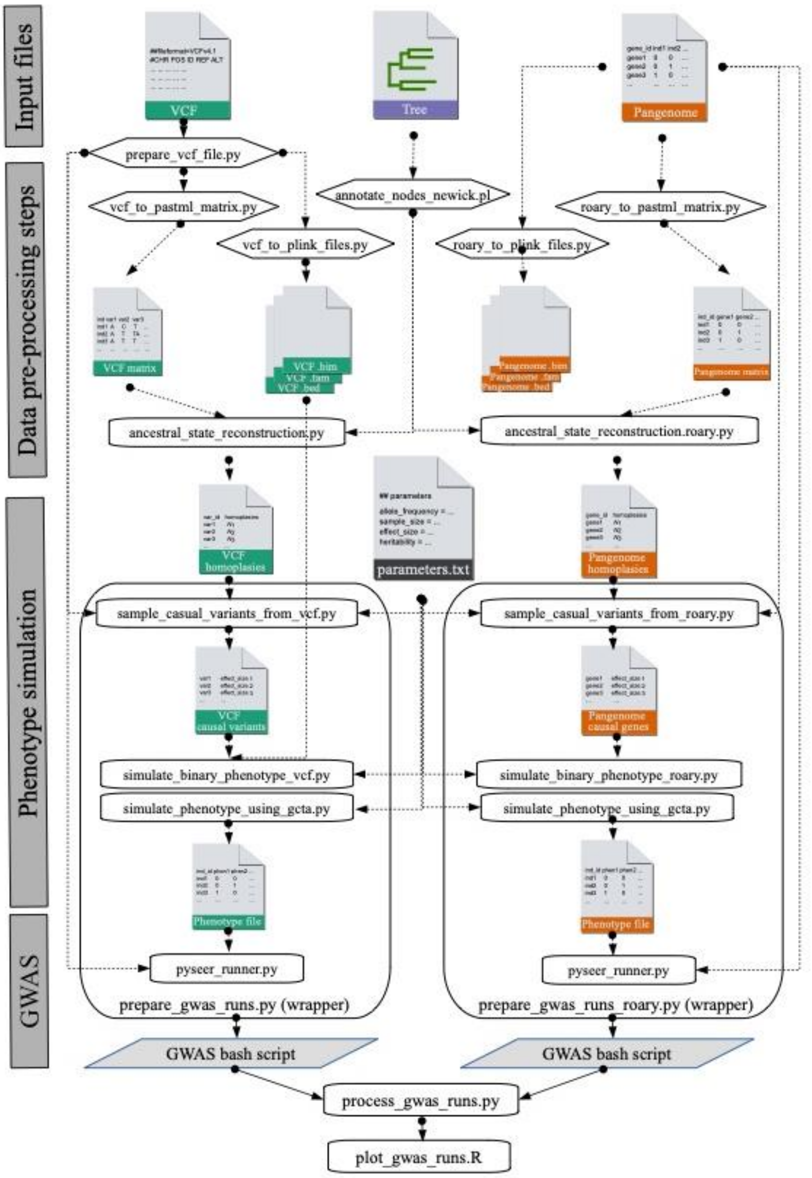

Input files, steps and scripts used to implement PowerBacGWAS. See 'Implementation of PowerBacGWAS pipeline' sections in Methods for details on when and what each script is used for.

Supplementary Table 1. Sample sizes required to detect AMR causal variants of different frequencies and effect sizes

Bacterial species (GWAS analysis)[1]	Strain collection (max. sample size)	Causal gene (max. freq.)	frequency[4]	Effect size (odds ratio)			
				small (1.5)	moderate (5)	large (10)	very large (100)
Enterococcus faecium (pan-genome GWAS)	species-wide (n=1,432)	Kanamycin resistance *aph(3')-IIIa* (56%)	1%	-	-	-	1000
			2.5%	-	-	800	700
			5%	-	800	600	400
			10%	-	500	300	200
	single-clade (n=761)[2]	Streptomycin resistance *ant(6)-Ia/aad(6)* (34%)	1%	-	-	-	-
			2.5%	-	-	-	700
			5%	-	-	700	400
			10%	-	700	400	200
Klebsiella pneumoniae (pan-genome GWAS)	species-wide (n = 2,628)[3]	Meropenem resistance bla_{KPC} (12%)	1%	-	-	-	-
			2.5%	-	-	1,400	1,000
			5%	-	1,100	800	600
			10%	-	700	500	300
Mycobacterium tuberculosis (burden GWAS)	species-wide (n = 2,655)	Isonizid resistance *katG* (20%)	1%	-	-	-	-
			2.5%	-	-	-	-
			5%	-	-	-	-
			10%	-	-	-	1,100
	single-clade (n = 1,139)	Isonizid resistance *katG* (13%)	1%	-	-	-	-
			2.5%	-	-	-	-
			5%	-	-	900	700
			10%	-	600	400	300

Results of running GWAS power calculations applying the *sub-sampling approach* for a binary phenotype and changing effect sizes (full heritability assumed). [1]Pan-genome GWAS was run for acquired AMR genes in *E. faecium* and *K. pneumoniae* populations. A burden testing GWAS was applied to *M. tuberculosis* populations to identify chromosomal genes with an enrichment of mutations in cases vs. controls. [2]The complete *E. faecium single-clade* collection (n=1,531) was not used because the streptomycin-resistance conferring gene was 79% frequent, too high to perform power calculations. [3]No results are shown for the *K. pneumoniae single-clade* population as no balanced proportion of meropenem-resistant and susceptible isolates were available. [4]'frequency' refers to the proportion of isolates carrying the acquired causal gene in *E. faecium* and *K. pneumoniae* populations; or proportion of isolates with known causal allele in the chromosomal AMR gene for *M. tuberculosis*. The maximum frequency tested could not be higher than that of AMR causal gene. -, non-detectable with 80% power.

Supplementary Table 2. Sample sizes required to detect causal genes of different frequencies and heritability values in a pan-genome GWAS

Bacterial species	Strain collection	gene frequency	Heritability (in percentage)			
			10%	25%	50%	99%
Enterococcus faecium (pan-genome GWAS)	species-wide (n=1,432)	1%	-	-	-	-
		2.5%	-	1,300	-	1,300
		5%	1,100	1,100	1,000	900
		10%	700	300	300	300
		25%	600	300	100	100
	single-clade (n=1,531)	1%	-	-	-	-
		2.5%	1,100	1,100	1,100	600
		5%	-	-	-	-
		10%	400	300	200	300
		25%	600	400	200	200
Klebsiella pneumoniae (pan-genome GWAS)	species-wide (n = 2,628)	1%	-	-	-	-
		2.5%	1,600	1,600	1,800	1,600
		5%	900	600	700	700
		10%	800	600	300	400
		25%	900	500	200	200
	single-clade (n = 1,193)	1%	-	-	-	-
		2.5%	-	-	-	-
		5%	-	1,000	1,000	1,000
		10%	1,100	1,000	800	900
		25%	1,000	600	400	400
Mycobacterium tuberculosis (pan-genome GWAS)	species-wide (n = 2,655)	1%	-	-	-	-
		2.5%	1,100	1,100	700	900
		5%	600	500	500	500
		10%	1,300	900	900	900
		25%	400	200	100	100
	single-clade (n = 1,139)	1%	-	-	-	-
		2.5%	1,000	1,000	1,000	900
		5%	600	500	400	400
		10%	400	200	200	200

		25%	700	400	200	300

This table shows the minimum sample sizes required to detect acquired genes of different heritability values (in percentage) and frequencies in a pan-genome GWAS (binary phenotype, effect size kept constant at an odds ratio of 2) with 80% power, in both species-wide and single-clade populations. Pan-genome GWAS was not run for *M. tuberculosis* populations. Abbreviations: -, non-detectable with 80% power.

Supplementary Table 3. Sample sizes required to detect SNPs of different MAF and heritability values

Bacterial species	Strain collection	MAF	variant GWAS			
			Heritability (in percentage)			
			10%	25%	50%	99%
E. faecium	species-wide (n=1,432)	1%	-	-	-	-
		2.5%	-	-	-	-
		5%	-	-	-	-
		10%	-	-	-	-
		25%	-	1,200	1,100	1,100
	single-clade (n=1,531)	1%	-	-	-	-
		2.5%	-	-	-	-
		5%	-	-	-	-
		10%	1,300	1,000	1,000	1,000
		25%	-	900	600	600
K. pneumoniae	species-wide (n = 2,628)	1%	-	-	-	-
		2.5%	-	-	-	-
		5%	1,900	1,600	1,600	1,300
		10%	1,400	700	600	800
		25%	1,500	600	300	300
	single-clade (n = 1,193)	1%	-	-	-	-
		2.5%	-	-	-	-
		5%	-	-	-	-
		10%	-	-	-	-
		25%	-	800	600	300
M. tuberculosis	species-wide (n = 2,655)	1%	-	-	-	-
		2.5%	-	-	-	-
		5%	-	-	-	-
		10%	-	-	-	-
		25%	-	1,400	600	600

	single-clade (n = 1,139)	0-1%	-	-	-	-
		2.5%	-	-	-	-
		5%	-	-	-	-
		10%	900	800	800	800
		25%	600	400	200	200

This table shows the minimum sample sizes required to detect acquired variants (i.e. mutations in the bacterial chromosome) of different heritability values (in percentage) and MAF using a variant or burden test GWAS (binary phenotype, effect size kept constant at an odds ratio of 2), in both species-wide and single-clade populations. Abbreviations: NA, no variants available with that MAF; -, non-detectable with 80% power.

Supplementary Table 4. Sample sizes required to detect causal SNPs of different MAF, effect sizes and number of homoplasies in a variant GWAS

			Effect size (odds ratio)											
Bacterial species	Strain collection	MAF	moderate (5)				large (10)				very large (100)			
			Number of homoplasies				Number of homoplasies				Number of homoplasies			
			1-5	5-10	10-50	50-100	1-5	5-10	10-50	50-100	1-5	5-10	10-50	50-100
E. faecium	species-wide (n=1,432)	2.5%	-	-	-	NA	-	-	-	NA	-	-	-	NA
		5%	-	-	-	1,300	-	-	-	800	-	-	900	600
		10%	-	-	-	700	1,000	1,300	800	500	600	700	500	300
		25%	-	1,000	700	400	1,000	500	400	200	400	300	300	100
	single-clade (n=1,531)	2.5%	-	-	-	NA	-	-	-	NA	-	1,400	1,200	NA
		5%	-	-	1,400	1,200	-	-	1,000	900	-	1,500	700	600
		10%	-	-	1,300	800	-	1,400	900	500	-	900	500	300
		25%	-	-	900	400	-	1,200	500	300	-	800	200	100
K. pneumoniae	species-wide (n = 2,628)	2.5%	-	-	-	2,500	-	2,500	2,200	1,600	-	2,400	1,500	1,200
		5%	1,400	-	2,000	1,500	1,100	1,900	1,300	1,000	500	1,500	900	500
		10%	800	700	900	800	500	500	600	500	300	300	400	400
		25%	NA	NA	300	400	NA	NA	200	200	NA	NA	100	100
	single-clade (n = 1,193)	2.5%	-	-	-	NA	-	-	-	NA	-	-	-	NA
		5%	-	-	-	NA	-	-	-	NA	-	-	-	NA
		10%	-	-	-	600	-	-	1,100	400	-	-	600	300
		25%	NA	-	700	NA	NA	700	400	NA	NA	400	200	NA
M. tuberculosis	species-wide (n = 2,655)	2.5%	-	-	-	-	-	-	-	-	-	-	-	1,400
		5%	-	-	-	-	-	-	-	-	-	-	-	-
		10%	-	-	-	-	-	-	-	-	-	-	-	-
		25%	-	-	-	800	-	-	-	500	-	-	-	200
	single-clade (n = 1,139)	2.5%	-	-	-	-	-	-	-	-	-	-	-	1,000
		5%	-	-	-	NA	-	-	-	NA	-	-	1,000	NA
		10%	-	-	900	500	-	-	900	400	-	-	600	300
		25%	NA	NA	NA	NA	NA	NA	NA	NA	NA	NA	NA	NA

This table shows the minimum sample sizes required to detect acquired variants (i.e. SNPs in the bacterial chromosome) of different MAF, effect sizes and number of homoplasies in a variant GWAS (binary phenotype, full heritability assumed), in both species-wide and single-clade populations. Abbreviations: MAF, minor allele frequency; NA, no variants available with that MAF and number of homoplasies; -, non-detectable with 80% power.
