# Supplementary material for: PowerBacGWAS: a computational pipeline to perform power calculations for bacterial genome-wide association studies
Source: Commun Biol. 2022 Mar 25;5:266. doi: 10.1038/s42003-022-03194-2 (PMC8956664; doi:10.1038/s42003-022-03194-2)
Supplement: Supplementary file 3 — Description of Additional Supplementary Files [file 42003_2022_3194_MOESM3_ESM.pdf]

Description of Additional Supplementary Files

File name: Supplementary Data 1
Description: Accessions code for the raw sequencing data.

File name: Supplementary Data 2
Description: Figure 2 source data.

File name: Supplementary Data 3
Description: Figure 3 source data.
