# Supplementary material for: PowerBacGWAS: a computational pipeline to perform power calculations for bacterial genome-wide association studies
Source: Commun Biol. 2022 Mar 25;5:266. doi: 10.1038/s42003-022-03194-2 (PMC8956664; doi:10.1038/s42003-022-03194-2)
Supplement: Supplementary file 7 — Reporting summary [file 42003_2022_3194_MOESM7_ESM.pdf]

nature research

Corresponding author(s): Francesc Coll

Last updated by author(s): Jan 15, 2021

Reporting Summary

Nature Research wishes to improve the reproducibility of the work that we publish. This form provides structure for consistency and transparency in reporting. For further information on Nature Research policies, see our Editorial Policies and the Editorial Policy Checklist.

Statistics

For all statistical analyses, confirm that the following items are present in the figure legend, table legend, main text, or Methods section.

n/a	Confirmed	
☐	☒	The exact sample size (*n*) for each experimental group/condition, given as a discrete number and unit of measurement
☒	☐	A statement on whether measurements were taken from distinct samples or whether the same sample was measured repeatedly
☐	☒	The statistical test(s) used AND whether they are one- or two-sided<br>*Only common tests should be described solely by name; describe more complex techniques in the Methods section.*
☐	☒	A description of all covariates tested
☐	☒	A description of any assumptions or corrections, such as tests of normality and adjustment for multiple comparisons
☒	☐	A full description of the statistical parameters including central tendency (e.g. means) or other basic estimates (e.g. regression coefficient) AND variation (e.g. standard deviation) or associated estimates of uncertainty (e.g. confidence intervals)
☒	☐	For null hypothesis testing, the test statistic (e.g. *F*, *t*, *r*) with confidence intervals, effect sizes, degrees of freedom and *P* value noted<br>*Give P values as exact values whenever suitable.*
☒	☐	For Bayesian analysis, information on the choice of priors and Markov chain Monte Carlo settings
☒	☐	For hierarchical and complex designs, identification of the appropriate level for tests and full reporting of outcomes
☐	☒	Estimates of effect sizes (e.g. Cohen's *d*, Pearson's *r*), indicating how they were calculated

Our web collection on statistics for biologists contains articles on many of the points above.

Software and code

Policy information about availability of computer code

Data collection	None applicable
Data analysis	The following open-source bioinformatics tools were used: Prokka v1.11, Roary v3.12.0, SMALT v0.1.19, SAMtools v0.1.19, snp-sites v2.5.1, Gubbins v1.4.10, RAxML v8.2.8, Kleborate v1.0.0, Snippy v4.6.0, FastTree v2.1.11, assembly_improvement pipeline (https://github.com/sanger-pathogens/assembly_improvement), BWA v0.7.17, GATK v4.1.4.1, iqtree v1.6.12, Pyseer v1.3.5, TBProfiler. The code related to the PowerBacGWAS pipeline can be found here: https://github.com/francesccoll/powerbacgwas.

For manuscripts utilizing custom algorithms or software that are central to the research but not yet described in published literature, software must be made available to editors and reviewers. We strongly encourage code deposition in a community repository (e.g. GitHub). See the Nature Research guidelines for submitting code & software for further information.

Data

Policy information about availability of data

All manuscripts must include a data availability statement. This statement should provide the following information, where applicable:

- Accession codes, unique identifiers, or web links for publicly available datasets
- A list of figures that have associated raw data
- A description of any restrictions on data availability

The whole genome sequences of the strain collections used in this study are available on the European Nucleotide Archive (ENA) under the accessions listed in Supplementary Data 1, which also includes the antibiotic susceptibility phenotypes and genotypes. The processed data (VCF, pan-genome, phylogenetic trees, phenotype, causal variants and parameters files) needed to run the PowerBacGWAS are available on GitHub (https://github.com/francesccoll/powerbacgwas/tree/main/data).

Field-specific reporting

Please select the one below that is the best fit for your research. If you are not sure, read the appropriate sections before making your selection.

☐ Life sciences ☐ Behavioural & social sciences ☒ Ecological, evolutionary & environmental sciences

For a reference copy of the document with all sections, see nature.com/documents/nr-reporting-summary-flat.pdf

Ecological, evolutionary & environmental sciences study design

All studies must disclose on these points even when the disclosure is negative.

Study description	Methodological study using existing collections of whole-genome sequenced bacterial strains
Research sample	For E. faecium, we used a strain collection isolated from a variety of sources in the UK of 1,432 isolate genomes from livestock and meat (n=256), wastewater treatment plants (n=383), bloodstream infections (n=782) and 11 NCTC strains. The E. faecium single-clade population consisted of 761 clade A1 isolates drawn from a haematology study, where the original population (of 1,477 isolates) was de-duplicated to retain a single isolate per subtype (strain type) and patient, and reduced down to 227 and 534 isolates from faecal and ward environmental sources, respectively. To increase the population of clade A1 genomes, we additionally included genomes from a UK hospital study (n=292) and a UK nation-wide (n=478) studies, which increased the population size to 1,531 clade A1 genomes.<br><br>For K. pneumoniae collection was assembled from seven studies and consisted of 2,628 isolates. The selected publications describe nation-wide or international isolate collections and include human, animal and environmental samples from Africa, Asia, the Caribbean and Europe. The K. pneumoniae single-clade population consisted of 239 CC258 isolates from this collection, plus 396 and 558 CC258 isolates from an extra two studies.<br><br>For M. tuberculosis, we chose a collection of clinical isolates (n=2,655) generated as part of a global drug resistance project2 and representing the four main human-infecting M. tuberculosis complex (MTBC) lineages (lineages 1 to 4). The M. tuberculosis single-clade population consisted of 1,139 isolates belonging to the most common sub-lineage (sub-lineage 4.3) in this global collection.
Sampling strategy	Three bacterial species were chosen to represent a gram-positive organism (E. faecium), a gram-negative (K. pneumoniae) and a species of limited genetic diversity (M. tuberculosis), respectively. For each species, a population representative of the species-wide strain diversity was obtained from available collections of whole-genome sequenced strains. A subset of this species-wide population with lower strain diversity, made up of samples from a single clade, was additionally selected (single-clade population).
Data collection	Not applicable, we used publicly available collections of whole-genome sequenced bacterial strains
Timing and spatial scale	Not applicable
Data exclusions	Not applicable, genomes of bad quality were already removed in original publications
Reproducibility	Not applicable
Randomization	Not applicable
Blinding	Not applicable

Did the study involve field work? ☐ Yes ☒ No

Reporting for specific materials, systems and methods

We require information from authors about some types of materials, experimental systems and methods used in many studies. Here, indicate whether each material, system or method listed is relevant to your study. If you are not sure if a list item applies to your research, read the appropriate section before selecting a response.

Materials & experimental systems

n/a	Involved in the study
☒	☐ Antibodies
☒	☐ Eukaryotic cell lines
☒	☐ Palaeontology and archaeology
☒	☐ Animals and other organisms
☒	☐ Human research participants
☒	☐ Clinical data
☒	☐ Dual use research of concern

Methods

n/a	Involved in the study
☒	☐ ChIP-seq
☒	☐ Flow cytometry
☒	☐ MRI-based neuroimaging
